# Intron variants of the p53 gene are associated with increased risk for ovarian cancer but not in carriers of BRCA1 or BRCA2 germline mutations

**DOI:** 10.1038/sj.bjc.6690669

**Published:** 1999-09

**Authors:** S Wang-Gohrke, W Weikel, H Risch, D Vesprini, J Abrahamson, C Lerman, A Godwin, R Moslehi, O Olipade, J-S Brunet, E Stickeler, D G Kieback, R Kreienberg, B Weber, S A Narod, I B Runnebaum

**Affiliations:** Molecular Biology Laboratory Department of Obstetrics and Gynaecology, University of Ulm, Ulm, D-89075, Germany; University of Mainz, Mainz, D-55101, Germany; Department of Epidemiology & Public Health, Yale University, New Haven, CT 06510-8034, USA; The Centre for Research in Women's Health, University of Toronto, ON, M5G 1N8, Canada; Lombardi Cancer Centre, Georgetown University Medical Centre, Washington DC, 20007-4104, USA; Divisions of Basic Science & Population Science, Fox Chase Cancer Centre, Philadelphia, 19012, USA; Department of Medical Genetics, University of British Columbia, Vancouver,BC, V6H 3N1, Canada; Cancer Risk Clinic Department of Medicine, Chicago, IL, USA; Departments of Medicine and Genetics, University of Pennsylvania, Philadelphia, PA, 19104, USA

**Keywords:** ovarian cancer, *p53*, polymorphism, *BRCA*1, *BRCA*2, genetic susceptibility

## Abstract

Two biallelic polymorphisms in introns 3 and 6 of the *p53* gene were analysed for a possible risk-modifying effect for ovarian cancer. Germline DNA was genotyped from 310 German Caucasian ovarian cancer patients and 364 healthy controls. We also typed 124 affected and 276 unaffected female carriers with known deleterious *BRCA*1 or *BRCA*2 germline mutation from high-risk breast-ovarian cancer families. Genotyping was based on PCR and high-resolution gel electrophoresis. German ovarian cancer patients who carried the rare allele of the *Msp*I restriction fragment length polymorphism (RELP) in intron 6 were found to have an overall 1.93-fold increased risk (95% confidence internal (CI) 1.27–2.91) which further increased with the age at diagnosis of 41–60 years (odds ratio (OR) 2.71, 95% CI 1.10–6.71 for 41–50 and OR 2.44, 95% CI 1.12–5.28 for 51–60). The 16 bp duplication polymorphism in intron 3 was in a strong linkage to the *Msp*I RFLP. In *BRCA*1 or *BRCA*2 mutation carriers, no difference in allele frequency was observed for carriers affected or unaffected with ovarian cancer. Our data suggest that intronic polymorphisms of the *p53* gene modify the risk for ovarian cancer patients but not in carriers with *BRCA*1 or *BRCA*2 mutations. © 1999 Cancer Research Campaign


British Journal of Cancer (1999) **81**(1), 179–183
© 1999 Cancer Research Campaign
Article no. bjoc.1999.0669

### Intron variants of the p53 gene are associated with increased risk for ovarian cancer but not in carriers of BRCA1 or BRCA2 germline mutations

**S Wang-Gohrke[1], W Weikel[2], H Risch[3], D Vesprini[4], J Abrahamson[4], C Lerman[5], A Godwin[6], R Moslehi[7], O Olipade[8], J-S Brunet[4], E Stickeler[1], DG Kieback[1]*, R Kreienberg[1], B Weber[9], SA Narod[4] and IB Runnebaum[1]***

[1]Molecular Biology Laboratory, Department of Obstetrics and Gynaecology, University of Ulm, Ulm D-89075, Germany; [2]University of Mainz, Mainz D-55101, Germany; [3]Department of Epidemiology & Public Health, Yale University, School of Medicine, New Haven, CT 06510-8034, USA; [4]The Centre for Research in Women's Health, University of Toronto, Toronto, ON, M5G 1N8, Canada; [5]Lombardi Cancer Centre, Georgetown University Medical Centre, Washington, DC 20007-4104, USA; [6]Divisions of Basic Science & Population Science, Fox Chase Cancer Centre, Philadelphia, PA 19012, USA; [7]Department of Medical Genetics, University of British Columbia, Vancouver, BC V6H 3N1, Canada; [8]Cancer Risk Clinic, Department of Medicine, University of Chicago, Chicago, IL, USA; [9]Departments of Medicine and Genetics, University of Pennsylvania, Philadelphia, PA 19104, USA

**Summary** Two biallelic polymorphisms in introns 3 and 6 of the *p53* gene were analysed for a possible risk-modifying effect for ovarian cancer. Germline DNA was genotyped from 310 German Caucasian ovarian cancer patients and 364 healthy controls. We also typed 124 affected and 276 unaffected female carriers with known deleterious *BRCA*1 or *BRCA*2 germline mutation from high-risk breast-ovarian cancer families. Genotyping was based on PCR and high-resolution gel electrophoresis. German ovarian cancer patients who carried the rare allele of the *Msp*I restriction fragment length polymorphism (RELP) in intron 6 were found to have an overall 1.93-fold increased risk (95% confidence internal (CI) 1.27–2.91) which further increased with the age at diagnosis of 41–60 years (odds ratio (OR) 2.71, 95% CI 1.10–6.71 for 41–50 and OR 2.44, 95% CI 1.12–5.28 for 51–60). The 16 bp duplication polymorphism in intron 3 was in a strong linkage to the *Msp*I RFLP. In *BRCA*1 or *BRCA*2 mutation carriers, no difference in allele frequency was observed for carriers affected or unaffected with ovarian cancer. Our data suggest that intronic polymorphisms of the *p53* gene modify the risk for ovarian cancer patients but not in carriers with *BRCA*1 or *BRCA*2 mutations.

**Keywords:** ovarian cancer; *p53*; polymorphism; *BRCA*1; *BRCA*2; genetic susceptibility

At least eight intronic *p53* polymorphisms have been detected in human genomic *p53*. These include a VNTR (variable number tandem repeat) region (Hahn et al, 1993) and a *Hae*III restriction fragment length polymorphism (RELP) (Ito et al, 1994) in intron 1, a G-to-C transversion in intron 2 (38 bp downstream of exon 2) (Pleasants and Hansen, 1994), a 16 bp duplication in intron 3 (5′-gacctggagggctggg-3′) (Lazar et al, 1993), a *Msp*I RFLP in intron 6 (G-to-A transition at 61 bp downstream of exon 6) (Peller et al, 1995; Mavridou et al, 1998), a G-to-C transversion at 37 bp upstream to exon 7 (Hillebrandt et al, 1997), an *Apa*I RFLP in intron 7 (Prosser and Condie, 1991), and an A-to-T transversion in intron 10 (Buller et al, 1995). Associations between cancer phenotypes and inherited *p53* intronic polymorphisms have been observed in studies on epithelial cancers, including ovarian, breast, colon, gastric, nasopharyngeal, thyroid, bladder and lung cancer (Birgander et al, 1995, 1996; Peller et al, 1995; Runnebaum et al, 1995; Sjalander et al, 1995, 1996; Hillebrandt et al, 1997; Mavridou et al, 1998; Wang-Gohrke et al, 1998*b*). In a previously reported clinic-based case-control study on the 16 bp duplication polymorphism in intron 3 we have found the rare allele to be significantly more frequent in ovarian cancer patients ($n = 62$) than in healthy controls (Runnebaum et al, 1995), this observation was not confirmed in two other studies on ovarian cancer patients (Lancaster et al, 1995; Campbell et al, 1996). Common variants of the *p53* intron sequences may represent low-penetrance germline mutations which affect *p53* gene function. The objectives of this study were to test whether germline *p53* polymorphisms in intron 3 and 6 modified the risk of ovarian cancer.

*Received 6 January 1999*
*Revised 11 February 1999*
*Accepted 23 February 1999*

*Correspondence to:* IB Runnebaum

### MATERIALS AND METHODS

### Case-control study on German ovarian cancer patients and healthy controls

A total of 310 ovarian cancer patients selected without regard to family history were identified who underwent surgery for histologically confirmed ovarian cancer at the University of Ulm from 1980 to 1995 and at the University of Mainz from 1980 to 1994. The age of the women ranged from 15 to 91 years with a mean age of 59 years. Blood samples were collected from 364 German female blood donors at the University of Ulm in 1993 to serve as controls. Blood donors had a history of no cancer and were unselected with respect to family history of cancer. Donors participated on a voluntary basis

*Present address: Department of Obstetrics and Gynaecology, University of Freiburg, 79106 Freiburg, Germany.

without financial incentives. All cases and controls were white German women from Southern and Central Germany. The average age of the controls was 41 years ranging from 17 to 65. Statistical adjustment for age was done as described below.

### *BRCA*1 and *BRCA*2 mutation carriers

A total of 400 carriers of deleterious *BRCA*1 or *BRCA*2 mutations were studied. The carriers originated from 212 breast-ovarian families in North America who were collected in the course of several epidemiological studies. There were 325 carriers of *BRCA*1 mutations and 75 carriers of *BRCA*2 mutations. One hundred and twenty-four of the 400 carriers had a history of ovarian cancer and 276 had no history of ovarian cancer. One hundred and twenty-seven of the carriers were Ashkenazi Jewish, including 79 of the women with ovarian cancer. The average age at the time of diagnosis of the ovarian cancer cases was 54.8 years compared to 47.6 years for the carriers without ovarian cancer. Both, affected and unaffected carriers came from the same families and so the ethnic distribution of the groups was similar.

### Ovarian cancer cell lines

Twenty-nine human ovarian cancer cell lines were established from primary ovarian cancer tissue as described previously (Mobus et al, 1992). The cells were grown in Dulbecco's modified Eagles medium (DMEM) containing 10% fetal calf serum, 200 mM glutamine, and 100 μg $ml^{-1}$ penicillin and 100 μg $ml^{-1}$ streptomycin in 5% carbon dioxide at 37°C and harvested at about 70% confluence.

### DNA extraction

Genomic DNA of cell lines and EDTA blood samples was extracted by the phenol chloroform method. For 158 cases, genomic DNA was isolated from the formalin-fixed paraffin-embedded uterine tissue unaffected by ovarian cancer by the sodium acetate precipitation method. Genomic DNA extraction for *BRCA*1 or *BRCA*2 carriers has been described elsewhere (Phelan et al, 1996).

### Genotyping

The analysis of the *p53* polymorphic sites was based on the polymerase chain reaction (PCR) amplification of two fragments encompassing the 16 bp duplication polymorphism in intron 3 and the *Msp*I RFLP in intron 6 (Wang-Gohrke et al, 1998*b*). All PCRs were carried out with 30 cycles each consisting of 1 min denaturing at 94°C, 1 min of annealing (60°C for intron 3, 54°C for intron 6) and 1 min of extension at 72°C. The amplification of the 16 bp duplication polymorphism using the sense primer OL-297 (5′-CTG AAA ACA ACG TTC TGG TA-3′) and antisense primer OL-298 (5′-AAG GGG GAC TGT AGA TGG GTG-3′) resulted in a 119 bp or 135 bp fragment. The 16 bp A1 allele was defined as the absence of the duplication, according to previous publications (Lazar et al, 1993; Runnebaum et al, 1995). The *Msp*I RFLP sense primer OL-301 (5′-TGG ATG ACA GAA ACA CTT TTC G-3′) and the antisense primer OL-302 (5′-GTG GTA CAG TCA GAG CCA ACC-3′) resulted in either a 152 bp fragment which after *Msp*I digest produced a 100 bp and a 52 bp fragment or in a 152 bp fragment which contained no *Msp*I cutting site. The PCR products were directly or following restriction enzyme digest run on a high resolution Hydrolink gel (AT Biochem, Malvern, USA) and processed by the software Fragment Manager in the automated sequencer ALF Express (Pharmacia Uppsala, Sweden). All individuals with a genotype containing the rare alleles heterozygously or homozygously were reanalysed starting from the DNA sample.

### Genomic DNA and cDNA sequencing of the *p53* gene

Genomic DNA of all 29 ovarian cancer cell lines was sequenced from exon 4 to exon 9 according to an established protocol (Wang-Gohrke et al, 1998*a*). For cDNA sequencing, total RNA was isolated from the cells by TRlzol® Reagent (GibcoBRL, Life Technologies, Inc, New York, USA). The first strand cDNA was synthesized by the RNAase $H^-$ Reverse Transcriptase directed by Oligo$(dT)_{12–18}$ Primer (GibcoBRL). Three primer pairs were designed to amplify three overlapping fragments which covered the entire cDNA sequence of *p53*. M13 forward and reverse primer sequences were attached to the 5′ terminus of the sense or antisense primer which allowed the sequencing of the PCR-fragments using M13 forward or reverse primers. The sequences of the three primer pairs were the following: p53cDNA1s: 5′-CCG GGG ACA CTT TGC GTT CG-3′, p53cDNA1as: 5′-TGA TTC CAC ACC CCC GCC C-3′; p53cDNA2s: 5′-ACT CCC CTG CCC TCA ACA AGA TG-3′, p53cDNA2as: 5′-AGC ACT AAG CGA GCA CTG CCC A-3′; p53cDNA3s: 5′-ACC GGC GCA CAG AGG AAG AGA A-3′, p53cDNA3as: 5′-CAT TTG CTT TGT CCC GGG GCT-3′. Annealing temperature for fragment 1 and 2 was 62°C and 64°C for fragment 3. PCR and cycle sequencing reactions have been described previously (Wang-Gohrke et al, 1998a). Cy5′-labelled (Cyanide) M13 forward and reverse primers were used in cycle sequencing. The products were analysed on a 6% denaturing acrylamide sequencing gel on an ALF Express Sequencer (Pharmacia Biotech, Uppsala, Sweden).

### Statistics

The association between 16 bp duplication polymorphism or *Msp*I RFLP and ovarian cancer was first estimated by comparing the proportions of women affected and unaffected by ovarian cancer, who carried the 16 bp A2 or the *Msp*I A1 allele. Statistical significance of the different proportions was measured by $\chi^2$ test. Yate's correction was used to identify deviations from Hardy–Weinberg proportions among cases and controls. Logistic regression models and stratified analysis for the case-control analysis controlling for age were used to estimate odds ratios (OR) and 95% confidence intervals (CI). Differences in genotypes as well as allele frequencies are given as Δ-values. The odds ratio for ovarian cancer in *BRCA*1 or *BRCA*2 carriers with or without the rare allele of *p53* polymorphisms was estimated using a Mantel–Haenszel approach. Significant associations were identified at the $P < 0.05$ level. All computations were performed using SAS, release 6.12 (SAS Institute, Carey, NC, USA).

### RESULTS

### Genotype analysis in German ovarian cancer patients and controls

Genotype analysis was performed for two polymorphic sites in the *p53* gene of 310 German women with ovarian cancer and 364

© Cancer Research Campaign 1999

**Table 1** *p53* genotypes and allele frequencies in ovarian cancer patients and healthy controls

| Polymorphism | Group | *n* | p53 | Genotype | | Allele | Freq. | $\chi^2$HW | OR[e] | 95% CI |
|---|---|---|---|---|---|---|---|---|---|---|
| | | | 1–1 | 1–2 | 2–2 | A1 | A2 | | | |
| 16 bp | Controls | 364 | 297 | 82 | 3 | 0.88 | 0.12 | 0.85 | | |
| | Cases[c] | 310 | 207 | 95 | 8 | 0.82 | 0.18[a] | 0.34 | 1.71 | 1.17–2.49 |
| *Msp*I RFLP | Controls | 364 | 3 | 73 | 288 | 0.11 | 0.89 | 0.20 | | |
| | Cases[d] | 310 | 7 | 92 | 211 | 0.17[b] | 0.83 | 0.42 | 1.93 | 1.27–2.91 |

Differences of allele frequencies: Chi-square test, df = 1; Cases vs Controls [a]for 16 bp, p = 0.003; [b]for *Msp*I, *P* = 0.001. Differences of genotype distributions: Chi-square test, df = 1; rare allele carriers vs common allele carriers: [c]16 bp (A2A2 + A1A2) vs A1A1: *P* = 0.004; [d]*Msp*I (A1A1 + A1A2) vs A2A2: *P* = 0.001. $\chi^2$HW: Chi-square values testing deviation from Hardy-Weinberg proportions (Yate's correction; df = 1). [e]Age-adjusted odds ratio.

healthy control women. Overall, the intron 6 *Msp*I rare allele (A1 allele) was found to be significantly associated with ovarian cancer (*P* = 0.001, Table 1) in German women with an increased odds ratio (OR adjusted for age 1.93, 95% CI 1.27–2.91). When analysing cases and controls by age groups, the odds ratios for *Msp*I A1 allele were found significantly high only in the patients with an age at diagnosis of 41–60 (OR 2.71, CI 1.10–6.71 for 41–50 and OR 2.44, CI 1.12–5.28 for 51–60). The 16 bp duplication polymorphism was found to be in a strong linkage to the *Msp*I RFLP. This linkage was observed in 287/310 (92.6%) ovarian cancer cases and in 355/364 (97.5%) controls. The age-adjusted odds ratio for the intron 3 16 bp rare allele (A2 allele) was 1.71, 95% CI 1.17–2.49 (*P* = 0.003). For 16 bp duplication polymorphism, the OR was significantly high only in the patients with an age at diagnosis of 51–60 (OR 2.2, CI 1.03–4.7). The genotype distributions in patients and controls did not deviate from expected Hardy–Weinberg frequencies at either site (Table 1).

At both polymorphic sites, the heterozygotes were more frequent in the ovarian cancer patients than the controls (for *Msp*I A1A2, Δ = 10%; for 16 bp A1A2, Δ = 8%). Among the ovarian cancer cases, the genotypes which were homozygous for the common allele (for *Msp*I A2A2, Δ = 11%; for 16 bp A1A1, Δ = 10%) were less frequent (Table 1). For both polymorphisms, the number of carriers of the rare allele was significantly higher in ovarian cancer patients (*P* = 0.004 for 16 bp duplication, *P* = 0.001 for *Msp*I RFLP).

For each individual, combined genotypes of both polymorphisms were analysed. A significant difference was found between patients and controls when the combination of common homozygotes (16 bp A1A1-*Msp*I A2A2) was compared with either the remaining genotypes (*P* = 0.001, Table 2) or the double heterozygotes (*P* = 0.005). Overall, double heterozygotes of the two polymorphisms were significantly more frequent in the ovarian cancer patients (26.5%) than in the control group (20.1%) with an age-adjusted OR of 1.65 (95% CI 1.07–1.11). This increased odds ratio was found to be significant only in the patients with an age at diagnosis of 51–60 (OR 2.3, CI 1.05–5.06). The common homozygote combination 16 bp A1A1-*Msp*I A2A2 was found with a higher frequency in the control group (Δ = 12.4%) resulting in a decreased age-adjusted OR of 0.52 (95% CI 0.35–0.77).

### Genotype analysis of *BRCA*1 or *BRCA*2 mutation carriers

Overall, the 16 bp A2 allele was seen in 25.8% of the carriers. The frequency was not significantly different for Jewish carriers (29.1%) and for non-Jewish carriers (24.2%). The frequency of the *p53* 16 bp duplication variant was not higher in the carriers with ovarian cancer (26.6%) than in carriers without ovarian cancer (25.4%). The odds ratio for ovarian cancer among those with the 16 bp A2 allele was 1.07 (95% CI 0.66–1.37). This odds ratio was higher for *BRCA*1 carriers (OR = 1.24) than for *BRCA*2 carriers (OR = 0.70) but neither of these ORs were statistically significant. Furthermore, there was no difference between the age of onset of ovarian cancer in the carriers with and without the 16 bp duplication; the average age at diagnosis of ovarian cancer for the 33 cases with the A2 allele was 55.0 years and the average age for the 91 cases without the A2 allele was 54.7 years. In the *BRCA*1 or *BRCA*2 carriers, the 16 bp duplication polymorphism in intron 3 was also found to be in a strong linkage to the *Msp*I RFLP in intron 6. Overall, there was no association between *p53* polymorphism status and the presence of ovarian cancer in individuals with germline mutations in *BRCA*1 or *BRCA*2.

**Table 2** Genotype combinations of the *p53* polymorphisms in ovarian cancer cases and healthy controls

| 16 bp | *Msp*I RFLP | Cases | Controls | OR[a] | 95% CI |
|---|---|---|---|---|---|
| 1–1 | 2–2 | 199 | 279 | 0.52[b] | 0.35–0.77 |
| 2–1 | 2–1 | 82 | 73 | 1.65[c,d] | 1.07–1.11 |
| 1–1 | 2–1 | 8 | 0 | | |
| 2–2 | 1–1 | 6 | 3 | 2.18[c] | 0.86–5.52 |
| 2–1 | 2–2 | 12 | 9 | 1.66[c] | 0.59–4.63 |
| 2–2 | 2–1 | 2 | 0 | | |
| 2–1 | 1–1 | 1 | 0 | | |
| Total | | 310 | 364 | | |

[a]Age-adjusted odds ratio. Cases vs Controls: 16 bp A1A1-*Msp*I A2A2, [b]vs the remainder of the combinations, *P* = 0.001; [d]vs 16 bp A1A2-*Msp*I A1A2, *P* = 0.005 (Chi-square test, df = 1). [c]All were compared with the combination of 16 bp A1A1-*Msp*I A2A2.

### Ovarian cancer cell lines

Twenty-nine cell lines were typed for both polymorphisms. The rare allele of the *Msp*I RFLP was in linkage with the rare allele of the 16 bp duplication polymorphism except for one cell line. For both polymorphisms, 25 cell lines were identified as homozygous for the common alleles (16 bp A1A1-*Msp*I A2A2), two as homozygous for the rare alleles (16 bp A1A1-*Msp*I A2A2) and one cell line was heterozygous. One other cell line showed a heterozygous 16 bp duplication polymorphism and a homozygous common allele of *Msp*I RFLP (OV-MZ-33). No splicing error was found when the *p53* sequencing was applied to the entire cDNA.

© Cancer Research Campaign 1999 

No mutation was identified in the 5′ or 3′ splice sites by genomic DNA sequencing of the flanking introns. In two cell lines with the genotype of 16 bp A1A1-*Msp*I A2A2, an A to G transition mutation at codon 205 of *p53* changed the amino acid sequence from a tyrosine to a cysteine in the OV-UL-02 cell line. The other cell line OV-MZ-22 had a wild-type sequence in cDNA and genomic DNA analysis.

### DISCUSSION

This study shows that germline variants of intron sequences of the *p53* gene or linked genetic variations may play a role in the development of sporadic ovarian cancer. The rare allele of *Msp*I RFLP was found to be associated with a more than twofold increase of risk in an age-dependent manner (OR 2.71, 95% CI 1.10–6.71 for 41–50 and OR 2.44, 95% CI 1.12–5.28 for 51–60). In an initial study on different cancer types, an increased number of *Msp*I RFLP heterozygotes were found in the germline DNA of gastrointestinal (GI) tumour patients (47.6%, $n = 21$, $P = 0.17$) and breast cancer patients (50%, $n = 20$, $P = 0.12$) comparing with a healthy control group (31.6%, $n = 38$) (Peller et al, 1995). A significant association was reported from a case-control study (65 vs 117) on American Caucasian breast cancer patients (Weston et al, 1997). The allele frequency of the rare *Msp*I RFLP was higher in cases (0.21) than in controls of the same ethnic background (0.11, $P = 0.02$). However, such an association was observed neither in our German (0.11 vs 0.15, $P = 0.075$) nor in an English case-control study (0.09 vs 0.10, $P = 0.88$) (Mavridou et al, 1998; Wang-Gohrke et al, 1998*b*). The comparison of the different studies is legitimate since the frequencies of the rare *Msp*I allele were similar in the Caucasian healthy control individuals in the American (0.11, $n = 117$), the English (0.10, $n = 254$) and German (0.11, $n = 305$) studies. It is possible that the significant association in the American Caucasian case-control study might be due to the specific ethnicity, the environment or the smaller size of the cohort genotyped (Weston et al, 1997). Another study on the same polymorphism in a Swedish population with and without breast cancer (212 vs 689) (Sjalander et al, 1996) could not be directly compared with other studies since the majority of the controls in this Swedish study consisted of placental samples. Another study which did not provide information on age found an association of this polymorphism with ovarian cancer (0.16 vs 0.10, $P = 0.01$) (Mavridou et al, 1998). In our study, this polymorphism did not appear to play a role in ovarian cancer development below the age of 40 (early-onset ovarian cancer).

We found the rare allele of the *Msp*I RFLP in a strong linkage with the rare allele of the 16 bp duplication polymorphism in controls (97.5%) as well as in ovarian cancer cases (92.6%). Therefore, it was not surprising to find that the genotype distribution of the 16 bp duplication polymorphism and its overall estimated relative risk for ovarian cancer (OR adjusted for age 1.71, 95% CI 1.17–2.49) were similar to those of the *Msp* I RFLP. In a previous pilot study, we have reported that the rare allele of the 16 bp duplication polymorphism intron 3 was significantly more frequent in a small group of German ovarian cancer patients (0.27, $n = 62$, $P = 0.001$) than in healthy controls (0.14, $n = 424$) (Runnebaum et al, 1995). This difference was observed neither in a British (0.16 vs 0.15) nor in an American (0.09 vs 0.13) case-control study (216 vs 113 and 82 vs 100 respectively) on Caucasian ovarian cancer patients (Lancaster et al, 1995; Campbell et al, 1996). Our current study confirms the higher prevalence of the rare allele in the cases as previously reported, even though the high frequency of homozygotes of the rare allele in our previous group was not representative (Runnebaum et al, 1995). Factors such as age, ethnicity and environment may have contributed to results differing from ours. No information on age was provided by the British and the American group.

Hereditary ovarian cancer can occur in the familial breast-ovarian cancer syndrome or in the hereditary non-polyposis colorectal cancer (HNPCC) syndrome which can include cancers of the colon, endometrium, breast or urinary tract (Lynch et al, 1991, 1993). The majority (81%) of the breast-ovarian cancer families are due to *BRCA*1, with most others (14%) due to *BRCA*2 (Ford et al, 1998). By the age of 70, the recently estimated risk of breast cancer among *BRCA*1 and *BRCA*2 carriers was 56% and of ovarian cancer 16% (Struewing et al, 1997). It has been hypothesized that the penetrance of *BRCA*1 or *BRCA*2 mutations could be modified by genetic and non-genetic factors. *HRAS*1 variable number of tandem repeat (VNTR) locus was identified to have an effect as a modifying gene variant on the penetrance of *BRCA*1 mutations for ovarian cancer (Phelan et al, 1996). In our case-control study of ovarian cancer among 400 women who have been identified to be carriers of a deleterious mutation in either the *BRCA*1 or the *BRCA*2 gene, the allelic frequencies of the two intronic *p53* polymorphisms did not significantly differ. No effect was found for the age of onset when comparing the carriers with and without the rare allele of the *p53* polymorphisms. This suggests that the intronic polymorphisms of the *p53* gene did not modify the risk to develop ovarian cancer beyond the effect of *BRCA*1 or *BRCA*2 mutations. The risk modification of *p53* polymorphisms appeared to be limited to sporadic cases.

*p53* introns have been shown to be implicated in regulation of gene expression and DNA–protein interaction (Hinds et al, 1990; Beenken et al, 1991; Lozano and Levine, 1991; Shamsher and Montano, 1996; Avigad et al, 1997). Single base pair substitutions in intron 4 have been shown to disturb binding of unidentified proteins resulting in a decreased expression of p53 (Beenken et al, 1991). Avigad et al (1997) identified a germ line variant in intron 6 (G-to-A substitution at 39 bp upstream of exon 7) in six paediatric patients with diverse tumours, all of which belonged to the Li–Fraumeni syndrome. High p53 protein expression was observed by immunohistochemistry (anti-p53 antibody DO-1) in the two available tumours and in one normal lymph node which carried a wild-type *p53* sequence in the coding region. The authors concluded that this intron mutation either caused or was linked with a variation leading to stabilization and possible inactivation of the p53 protein thereby contributing to neoplastic transformation (Avigad et al, 1997). In our study, a medium or low p53 expression was observed in three wild-type *p53* cell lines with the rare allele of both *p53* intronic polymorphisms. The immunostaining did not differ from the ICC pattern of 13 other cell lines with wild-type *p53* without the rare allele. As analysed by sequencing of genomic DNA and cDNA, mutations in the coding region are not necessarily linked to the polymorphisms. As demonstrated by the cell line with a *p53* mis-sense mutation, the two polymorphisms did not seem to be sufficient to impair p53 function during the selection process in tumorigenesis but needed an additional coding region mutation. No splicing errors were found to be linked to the polymorphisms. The data presented here encourage to further study the possible mechanisms by which the germline intron 3 and intron 6 variants modify cancer risk and to search for closely linked genetic aberrations in neighbouring genes.

© Cancer Research Campaign 1999

### ACKNOWLEDGEMENTS

The authors wish to thank Ms Sabine Hees for expert technical assistance and Dr Werner Koerner for providing buffy coats from the Blood Bank, University of Ulm. This study was funded by the Deutsche Forschungsgemeinschaft (DFG RU476/2-1) and an institutional fund (P.460) by the state of Baden-Württemberg granted to IBR through the Klinikumsvorstand.

### REFERENCES

Avigad S, Barel D, Blau O, Malka A, Zoldan M, Mor C, Fogel M, Cohen IJ, Stark B, Goshen Y, Stein J and Zaizov R (1997) A novel germline p53 mutation in intron 6 in diverse childhood malignancies. *Oncogene* **14**: 1541–1545

Beenken SW, Karsenty G, Raycroft L and Lozano G (1991) An intron binding protein is required for transformation ability of p53. *Nucleic Acids Res* **19**: 4747–4752

Birgander R, Sjalander A, Rannug A, Alexandrie AK, Sundberg MI, Seidegard J, Tornling G, Beckman G and Beckman L (1995) P53 polymorphisms and haplotypes in lung cancer. *Carcinogenesis* **16**: 2233–2236

Birgander R, Sjalander A, Zhou Z, Fan C, Beckman L and Beckman G (1996) p53 polymorphisms and haplotypes in nasopharyngeal cancer. *Hum Hered* **46**: 49–54

Buller RE, Skilling JS, Kaliszewski S, Niemann T and Anderson B (1995) Absence of significant germ line p53 mutations in ovarian cancer patients. *Gynecol Oncol* **58**: 368–374

Campbell IG, Eccles DM, Dunn B, Davis M and Leake V (1996) p53 polymorphism in ovarian and breast cancer. *Lancet* **347**: 393–394

Ford D, Easton DF, Stratton M, Narod S, Goldgar D, Devilee P, Bishop DT, Weber B, Lenoir G, Chang Claude J, Sobol H, Teare MD, Struewing J, Arason A, Scherneck S, Peto J, Rebbeck TR, Tonin P, Neuhausen S, Barkardottir R, Eyfjord J, Lynch H, Ponder BA, Gayther SA, Zelada Hedman M and the Breast Cancer Linkage Consortium (1998) Genetic heterogeneity and penetrance analysis of the *BRCA*1 and *BRCA*2 genes in breast cancer families. The Breast Cancer Linkage Consortium. *Am J Hum Genet* **62**: 676–689

Hahn M, Serth J, Fislage R, Wolfes H, Allhoff E, Jonas V and Pingoud A (1993) Polymerase chain reaction detection of a highly polymorphic VNTR segment in intron 1 of the human *p53* gene. *Clin Chem* **39**: 549–550

Hillebrandt S, Streffer C, Demidchik EP, Biko J and Reiners C (1997) Polymorphisms in the *p53* gene in thyroid tumours and blood samples of children from areas in Belarus. *Mutat Res* **381**: 201–207

Hinds PW, Finlay CA, Quartin RS, Baker SJ, Fearon ER, Vogelstein B and Levine AJ (1990) Mutant p53 DNA clones from human colon carcinomas cooperate with ras in transforming primary rat cells: a comparison of the ‘hot spot’ mutant phenotypes. *Cell Growth Differ* **1**: 571–580

Ito T, Seyama T, Hayashi T, Mizuno T, Iwamoto KS, Tsuyama N, Dohi K, Nakamura N and Akiyama M (1994) Hae III polymorphism in intron 1 of the human *p53* gene. *Hum Genet* **93**: 222

Lancaster JM, Brownlee HA, Wiseman RW and Taylor J (1995) p53 polymorphism in ovarian and bladder cancer. *Lancet* **346**: 182

Lazar V, Hazard F, Bertin F, Janin N, Bellet D and Bressac B (1993) Simple sequence repeat polymorphism within the *p53* gene. *Oncogene* **8**: 1703–1705

Lozano G and Levine AJ (1991) Tissue-specific expression of p53 in transgenic mice is regulated by intron sequences. *Mol Carcinog* **4**: 3–9

Lynch HT, Watson P, Bewtra C, Conway TA, Hippee CR, Kaur P, Lynch JF and Ponder BA (1991) Hereditary ovarian cancer. Heterogeneity in age at diagnosis. *Cancer* **67**: 1460–1466

Lynch HT, Smyrk TC, Watson P, Lanspa SJ, Lynch JF, Lynch PM, Cavalieri RJ and Boland CR (1993) Genetics, natural history, tumor spectrum, and pathology of hereditary non-polyposis colorectal cancer: an updated review. *Gastroenterology* **104**: 1535–1549

Mavridou D, Gornall R, Campbell IG and Eccles DM (1998) TP53 intron 6 polymorphism and the risk of ovarian and breast cancer. *Br J Cancer* **77**: 676–677

Mobus V, Gerharz CD, Press U, Moll R, Beck T, Mellin W, Pollow K, Knapstein PG and Kreienberg R (1992) Morphological, immunohistochemical and biochemical characterization of 6 newly established human ovarian carcinoma cell lines. *Int J Cancer* **52**: 76–84

Peller S, Kopilova Y, Slutzki S, Halevy A, Kvitko K and Rotter V (1995) A novel polymorphism in intron 6 of the human *p53* gene: a possible association with cancer predisposition and susceptibility. *DNA Cell Biol* **14**: 983–990

Phelan CM, Rebbeck TR, Weber BL, Devilee P, Ruttledge MH, Lynch HT, Lenoir GM, Stratton MR, Easton DF, Ponder BA, Cannon Albright L, Larsson C, Goldgar DE and Narod SA (1996) Ovarian cancer risk in BRCA1 carriers is modified by the HRAS1 variable number of tandem repeat (VNTR) locus. *Nat Genet* **12**: 309–311

Pleasants LM and Hansen MF (1994) Identification of a polymorphism in intron 2 of the *p53* gene. *Hum Genet* **93**: 607–608

Prosser J and Condie A (1991) Biallelic ApaI polymorphism of the human *p53* gene (TP53). *Nucleic Acids Res* **19**: 4799

Runnebaum IB, Tong XW, Konig R, Zhao H, Korner K, Atkinson EN, Kreienberg R, Kieback DG and Hong Z (1995) p53-based blood test for p53pin3 and risk for sporadic ovarian cancer. *Lancet* **345**: 994

Shamsher M and Montano X (1996) Analysis of intron 4 of the p53 gene in human cutaneous melanoma. *Gene* **176**: 259–262

Sjalander A, Birgander R, Athlin L, Stenling R, Rutegard J, Beckman L and Beckman G (1995) *p53* germline haplotypes associated with increased risk for colorectal cancer. *Carcinogenesis* **16**: 1461–1464

Sjalander A, Birgander R, Hallmans G, Cajander S, Lenner P, Athlin L, Beckman G and Beckman L (1996) p53 polymorphisms and haplotypes in breast cancer. *Carcinogenesis* **17**: 1313–1316

Struewing JP, Hartge P, Wacholder S, Baker SM, Berlin M, McAdams M, Timmerman MM, Brody LC and Tucker MA (1997) The risk of cancer associated with specific mutations of BRCA1 and BRCA2 among Ashkenazi Jews. *N Engl J Med* **336**: 1401–1408

Wang-Gohrke S, Hees S, Pochon A, Wen WH, Reles A, Press MF, Kreienberg R and Runnebaum IB (1998*a*) Genomic semi-automated cycle sequencing as a sensitive screening technique for p53 mutations in frozen tumor samples. *Oncology Reports* **5**: 65–68

Wang-Gohrke S, Rebbeck TR, Besenfelder W, Kreienberg R and Runnebaum IB (1998*b*) P53 germline polymorphisms are associated with an increased risk for breast cancer in German women. *Anticancer Res* **18**: 2095–2100

Weston A, Pan CF, Ksieski HB, Wallenstein S, Berkowitz GS, Tartter PI, Bleiweiss IJ, Brower ST, Senie RT and Wolff MS (1997) p53 haplotype determination in breast cancer. *Cancer Epidemiol Biomarkers Prev* **6**: 105–112

© Cancer Research Campaign 1999 
